# A 44-Year-Old Male With Cerebral Venous Sinus Thrombosis

**DOI:** 10.7759/cureus.36974

**Published:** 2023-03-31

**Authors:** Talha Shabbir, Rachel Hunsucker, Daisy Martin, Zian Shabbir, Hadi Abou-El-Hassan, Tehmina Salahuddin

**Affiliations:** 1 Medical School, California University of Science and Medicine, Colton, USA; 2 Medical School, California Health Sciences University, Clovis, USA; 3 Neurology, Arrowhead Regional Medical Center, Colton, USA; 4 Neurology, California University of Science and Medicine, Colton, USA

**Keywords:** stroke, intracranial hemorrhage, headache, anticoagulation, cerebral venous sinus thrombosis (cvst)

## Abstract

Cerebral venous sinus thrombosis (CVST) is a rare condition that can result in severe neurological complications when left untreated. Disease pathology results from thrombus development within the superficial cortical veins or the dural sinuses. Thrombosis impedes cerebral drainage leading to venous congestion and consequent increase in cerebral pressure, parenchymal damage, and blood-brain barrier disruption. Headache is the most common presenting symptom; other symptoms include focal neurological signs, seizures, papilledema, and altered sensorium. Diagnosis is typically made with visualization of obstructed flow in the cerebral venous system using one of three imaging modalities: computed tomography-venography (CTV), magnetic resonance imaging with venography (MRV), and diagnostic cerebral angiography. First-line therapy for CVST is anticoagulation, and the prognosis is generally favorable with early detection and prompt treatment. In this case report, we discuss a singular case of a patient presenting with loss of consciousness who was found to have CVST and treated with anticoagulation therapy in the setting of an intraparenchymal hemorrhage.

## Introduction

Cerebral venous sinus thrombosis (CVST) is a rare condition with an approximate incidence rate of 1-2 per 100,000 person-years [1]. When left untreated, CVST can result in severe neurological complications, such as stroke, seizures, or coma [1-8]. Patients with underlying CVST account for 0.5-1% of all stroke cases [1,5]. From an epidemiology perspective, the disease typically occurs in middle-aged individuals, with a higher predilection for females to males, possibly due to higher estrogen levels during pregnancy or with hormonal contraceptive use [1,4-7]. Similar to other venous thromboembolic pathologies, CVST development can be attributed to various factors altering the balance between pro-thrombotic and thrombolytic mediators [7,9].

Within an overview of intracranial venous drainage, blood from cerebral capillary networks collects into superficial and deep cerebral veins. The superficial and deep venous systems converge into larger dural sinuses that eventually flow into the internal jugular vein [2,5-7]. In CVST, underlying pathology leads to thrombus development within the cerebral veins or the dural sinuses resulting in subsequent complications if left untreated [2,5-7]. Among these vessels, the two most common sites of thrombosis are the superior sagittal sinus (SSS) and the lateral (transverse) sinuses [6,7]. The development of a thrombus can be spontaneous or attributed to several acquired or genetic risk factors [1-7]. Acquired risk factors include malignancies, obesity, trauma, pregnancy, estrogen-containing contraceptives, and infections [1-7]. Genetic risk factors include immune-mediated diseases and thrombophilia such as Factor V Leiden, Protein C deficiency, Protein S deficiency, and Prothrombin gene mutations [1-7]. Ultimately, the formation of thrombus results in cerebral blood flow obstruction resulting in complications downstream and upstream from the blockage [2,4-6].

Complications of CVST can be attributed to two mechanisms: increased intracranial pressure (ICP) and ischemia [2,4-6]. Decreased drainage due to a venous thrombus causes venous congestion and an increase in ICP with consequent compressive injury and stasis of deoxygenated blood proximal to the thrombus resulting in poor oxygen delivery (ischemia) to tissue distal to the lesion [2,4-6]. Additionally, an increase in venous pressure and ischemia can result in infarcts with a high incidence of hemorrhagic transformation [4]. Symptoms classically seen with CVST are secondary to these mechanisms and vary based on thrombus location [1,3,5]. Headache is the primary symptom and often the only presenting complaint in patients with CVST [1-7]. On initial presentation, headaches were reported in roughly 70-90% of cases, with typically no additional neurological exam abnormalities [2,6,7]. Although less common, other symptoms include focal neurological signs, seizures, papilledema, or altered sensorium [2-7].

Advancements in non-invasive diagnostic imaging have improved early detection and prompt intervention for CVST. Due to its rarity, it is often under-recognized and requires a high index of suspicion for appropriate diagnostics [5]. Once diagnosed, the standard of care is anticoagulation therapy [1-10]. Fortunately, with adequate treatment, the prognosis for CVST is generally favorable, and most patients note improvements in symptoms with no long-term neurological deficits [4-7].

In this case report, we discuss a male patient presenting with loss of consciousness and found incidentally to have CVST with underlying intraparenchymal hemorrhage treated with anticoagulation.

## Case presentation

We present a case of a 44-year-old gentleman with a history of hypertension and dyslipidemia. He was an on-duty correctional officer escorting an inmate to the county hospital when he collapsed in the computed tomography (CT) scanner room. The event, witnessed by staff, was described as abrupt seizure-like activity, resulting in code stroke initiation. The patient spontaneously returned to consciousness within 1-2 minutes, followed by a 15-20-minute period of disorientation. Neurological examination in the emergency room (ER) noted a Glasgow Coma Score (GCS) of 15, no focal neurological signs, and the patient demonstrating postictal confusion. Otherwise, additional neurological testing, including sensory, motor, and cranial nerve examinations, were unremarkable.

**Figure 1:**
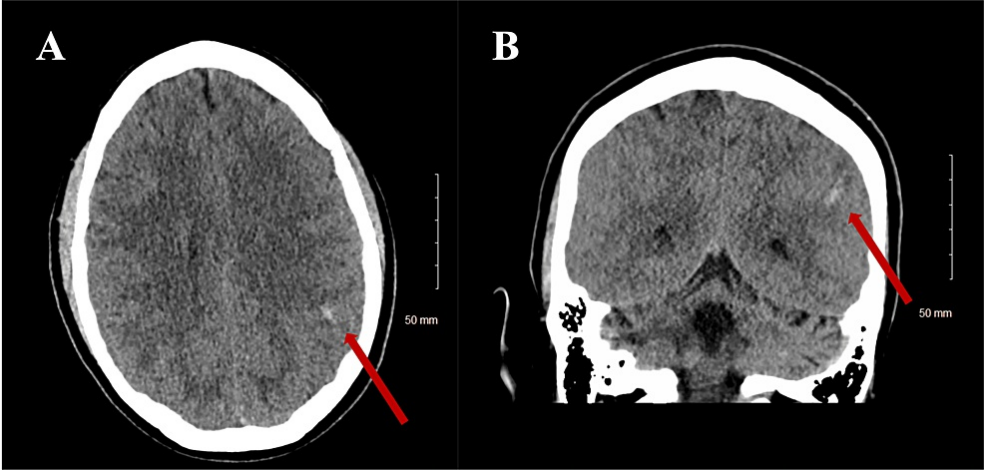
Non-Contrast CT of the Head (A) Axial non-contrast CT head showing one of the two foci of intraparenchymal hemorrhages. (B) Non-contrast CT head showing a small intraparenchymal hemorrhage on the coronal sequence. CT: computed tomography

Admission blood work and electrocardiogram were unremarkable. As part of the stroke workup, a non-contrast CT scan of the head revealed two small left parietal foci of intraparenchymal hemorrhage without ventricular extension, midline shift, or abnormalities of the basal cisterns (Figures 1A-1B).

**Figure 2:**
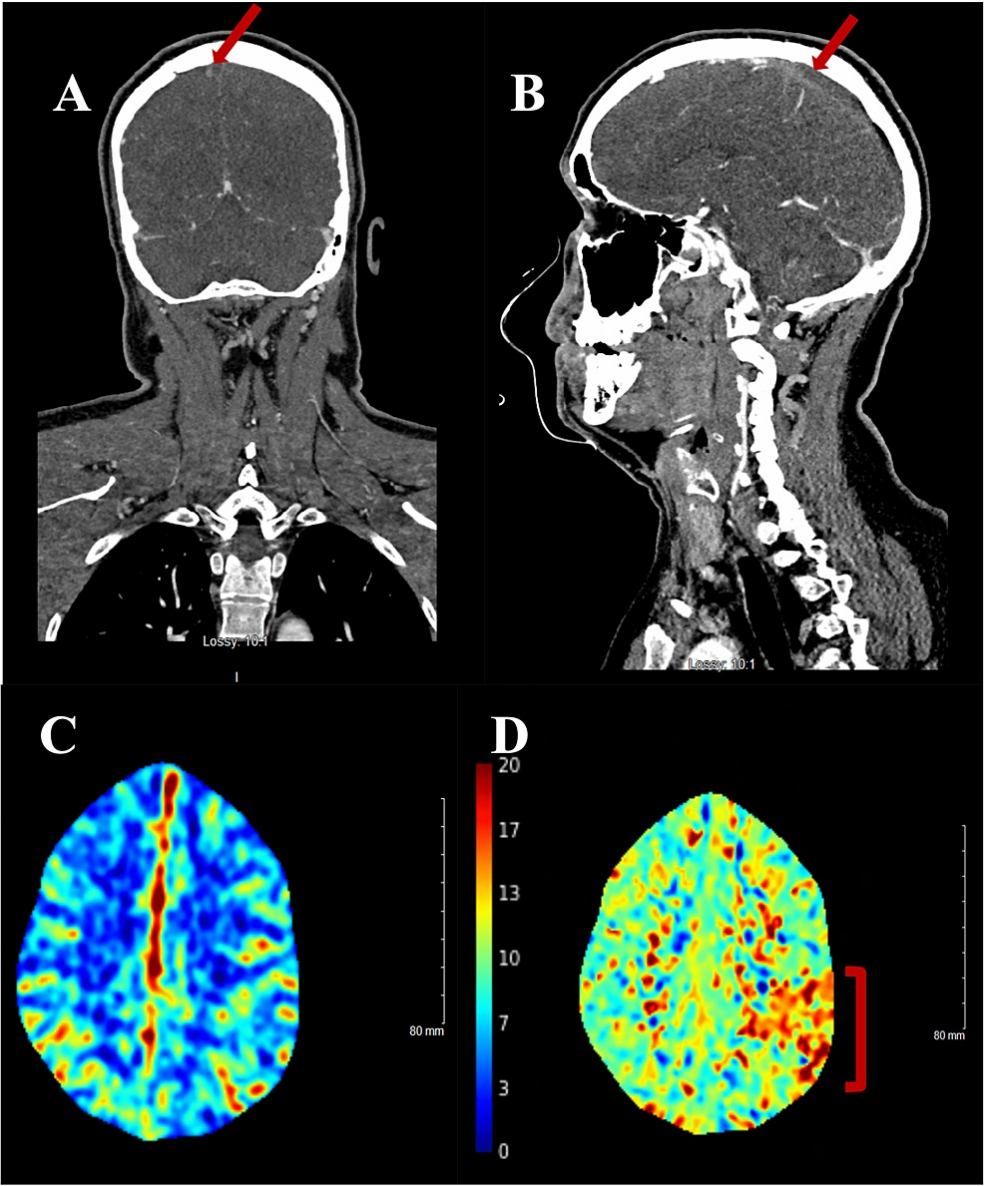
CT Angiogram and Perfusion Scans of the Head (A) Coronal and (B) Sagittal CTA demonstrating the absence of blood flow in the superior sagittal sinus. (C) Normal symmetric bilateral CBV on CT perfusion. (D) MTT sequence of the CT perfusion scan showing increased mean transit time in the left parietal lobe. CT: computed tomography; CTA: computed tomography angiogram; CBV: cerebral blood volume; MTT: mean transit time

Additional imaging with a CT angiogram of the head and neck with contrast revealed a flow defect in the SSS, indicating sinus thrombosis (Figures 2A-2B). CT perfusion study showed abnormal perfusion within the left parietal lobe without a core infarct and a volume mismatch of 6 milliliters (Figures 2C-2D).

**Figure 3:**
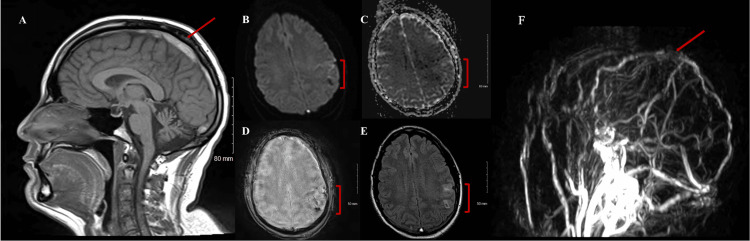
MRI and MRV of the Brain without Contrast (A) T1 Sagittal MRI of the brain showing thrombosis of the superior sagittal sinus. (B) Diffusion-weighted imaging and (C) apparent diffusion coefficient sequences of the brain MRI showing no restricted-diffusion. (D) T2 star and (E) FLAIR sequences of the brain MRI showing the intraparenchymal hemorrhages. (F) MRV showing thrombosis in the superior sagittal sinus. MRI: magnetic resonance imaging; MRV: magnetic resonance venography; FLAIR: fluid attenuated inversion recovery

Due to concern for heavy iodinated contrast load from multiple studies, the multidisciplinary care team made a choice for magnetic resonance (MR) over CT venography. The venogram demonstrated thrombosis in the posterior two-thirds of the SSS; two small foci of left parietal hemorrhage were also noted (Figure 3A-3E).

**Figure 4:**
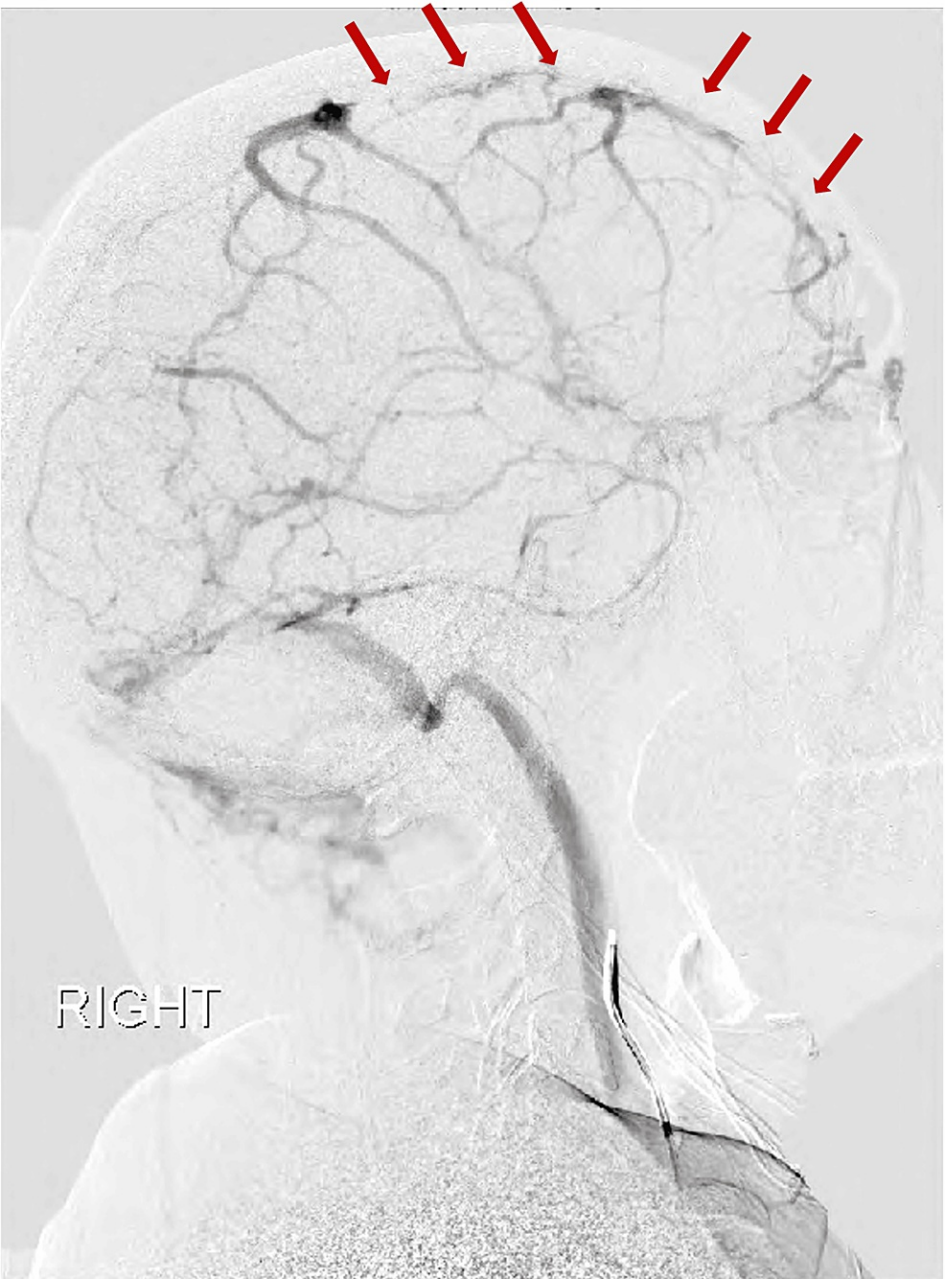
Diagnostic Cerebral Angiogram Diagnostic cerebral angiogram showing flow defect in the superior sagittal sinus.

The patient was admitted to the neuro-intensive care unit (Neuro-ICU) with heparin infusion for anticoagulation and phenytoin for seizure prophylaxis. The initial coagulopathy screen was unremarkable. Additionally, a confirmatory diagnostic cerebral angiogram, ordered to assess the extent of the disease burden, reconfirmed posterior SSS thrombosis with good venous return via multiple collaterals (Figure 4).

A subsequent repeat non-contrast CT head was stable. After two days of treatment, the care team transitioned therapy from heparin infusion to oral anticoagulation, and the patient was discharged after a full week in the inpatient ICU with a planned follow-up in the Neurology clinic. At the time of discharge, the exact etiology of the CVST was unclear as the workup for hypercoagulable states was thus far unremarkable. A more comprehensive workup was planned for the outpatient setting outside of the acute period.

## Discussion

Most patients with CVST present with headache as the primary and often sole presenting symptom [1-7]. Though the patient in our report eventually had a seizure, his primary initial symptom was a persistent, unremitting month-long headache for which he had not sought medical attention. Many cases of CVST remain unrecognized due to the variability and nonspecific nature of presenting symptoms such as headaches, which are a common complaint in numerous conditions such as migraine, meningitis, sinusitis, and hemorrhagic stroke, to name a few [3-6]. CVST may also present with focal neurological deficits or seizures, causing further variability and diagnostic challenges for providers [1-7].

Due to the nonspecific clinical presentation of CVST, a high index of suspicion is necessary for diagnosis and treatment. Diagnosis requires visualization of the obstructive lesion in the cerebral venous system using three non-invasive modalities: CT-V, MR-V, and diagnostic cerebral angiography [2-5]. MR-V is the most sensitive and has the advantage of showing masses, lesions, and intracranial hemorrhage, especially when paired with standard MRI, though MR-V alone is prone to motion artifact [3-7]. CT-V and diagnostic cerebral angiography are also noted to have high sensitivity, allowing for adequate diagnosis of CVST [3-7]. As part of the stroke workup, an emergent non-contrast CT head revealed two small foci of intraparenchymal hemorrhages. Additional workup with a CT angiogram of the head and neck first noted abnormal flow in the SSS, signifying the likely presence of sinus thrombosis. CT perfusion with wedge-shaped mismatch defect without any arterial lesion raised suspicion for a venous entity and further exploration with MR-V and diagnostic cerebral angiogram. Due to these findings, the parietal lobe hemorrhages were likely secondary to persistent sinus thrombosis. 

The first-line therapy for CVST is anticoagulation [1-10]. Of note, the presence of consequent hemorrhage is not a contraindication to treatment. Generally, low-molecular-weight heparin (LMWH) is preferred over unfractionated heparin (UFH), except in cases that may require surgical intervention where rapid reversal may be necessary [4,5]. Second-line treatments are considered on a case-by-case basis, as there is limited evidence of their efficacy. Cases that are more severe or refractory to anticoagulation may require an endovascular intervention such as mechanical thrombectomy and endovascular thrombolysis [1,2,4-7]. In addition to treating thrombosis, a decompressive craniotomy may be indicated in cases of mass effects from hemorrhagic or cytotoxic edema [1-5]. Acute neurocritical management includes fluid maintenance, hemodynamic stability, ICP monitoring, and MAP maintenance for optimal perfusion for the survival of preserved parenchyma [1-3].

Contraindications for the use of heparin include thrombocytopenia, a history of heparin-induced thrombocytopenia, and the presence of active, uncontrollable bleeding [11]. It is important to note that while it may seem counterintuitive to employ anticoagulation in a patient with demonstrated intracranial hemorrhage, heparin is the standard of care in CVST treatment due to its demonstrated efficacy and safety. The randomized control trials and retrospective studies by Einhäupl et al. found that heparin did not cause or worsen IPH when treating CVST and decreased mortality in patients with or without concomitant IPH. As such, Einhäupl et al. reported that the presence of IPH is not a contraindication to heparin use when treating CVST [10]. Heparin is, therefore, the standard of care, even with concurrent IPH [1,2,4-7,10].

Rapid detection and treatment over the past decades have improved morbidity and mortality of CVST, with mortality currently estimated between 5% and 10% [4,5]. When promptly detected and treated, CVST has a generally favorable prognosis with minimal persistent neurological complications; poor prognosticators are older age, GCS <9, seizures, intracranial hemorrhage (ICH), malignancy, and CNS infection [1,3,4,6,7].

## Conclusions

This case report presents a patient with prolonged unremitting headaches and seizures secondary to CVST. The patient's hospital course highlights the various imaging modalities employed to diagnose his venous sinus thrombosis and anticoagulation therapy. Although heparin can complicate hemorrhage, it is the established first-line treatment. Due to the rarity and nonspecific presentation of venous sinus thrombosis, a high index of suspicion is necessary to include it in differential diagnoses for patients with headaches in the right clinical setting to ensure prompt management and a favorable outcome.
